# Molecular hydrogen: an overview of its neurobiological effects and therapeutic potential for bipolar disorder and schizophrenia

**DOI:** 10.1186/2045-9912-3-11

**Published:** 2013-06-06

**Authors:** Ahmad Ghanizadeh, Michael Berk

**Affiliations:** 1 Research Center for Psychiatry and Behavioral Sciences, Shiraz University of Medical, Sciences, Hafez Hospital, Shiraz, Iran; 2 Department of Psychiatry, Hafez Hospital, School of Medicine, Shiraz University of Medical Sciences, Shiraz, Iran; 3 School of Medicine, Deakin University, Melbourne, Australia; 4 The Florey Institute of Neuroscience and Mental Health, Orygen Research Centre, University of Melbourne, 234 St Kilda Rd, Southbank VIC 3006, Australia; 5 The Department of Psychiatry, University of Melbourne, 234 St Kilda Rd, Southbank, VIC 3006, Australia

## Abstract

Hydrogen gas is a bioactive molecule that has a diversity of effects, including anti-apoptotic, anti-inflammatory and anti-oxidative properties; these overlap with the process of neuroprogression in major psychiatric disorders. Specifically, both bipolar disorder and schizophrenia are associated with increased oxidative and inflammatory stress. Moreover, lithium which is commonly administered for treating bipolar disorder has effects on oxidative stress and apoptotic pathways, as do valproate and some atypical antipsychotics for treating schizophrenia. Molecular hydrogen has been studied pre-clinically in animal models for the treatment of some medical conditions including hypoxia and neurodegenerative disorders, and there are intriguing clinical findings in neurological disorders including Parkinson’s disease. Therefore, it is hypothesized that administration of hydrogen molecule may have potential as a novel therapy for bipolar disorder, schizophrenia, and other concurrent disorders characterized by oxidative, inflammatory and apoptotic dysregulation.

## Introduction

Normally, there is a balance between oxidant and antioxidant systems in body. When there is an imbalance between anti-oxidative defenses and prevailing oxidative stress, reactive oxidative species are increased leading to inflammation and oxidative damage marked by protein carbonylation, DNA damage [1,2] and lipid peroxidation. These biomarkers are documented in all illness phases, but appear more pronounced in episodes of acute illness, particularly mania [3].

Bipolar mood disorder is a relatively common neuropsychiatric disorder with an estimated prevalence of 1% to 2% [4], and a high burden of disease [4]. There is a high rate of medical comorbidity including diabetes and the metabolic syndrome [5], cardiovascular morbidity [6], and obesity [7], all of which are associated with inflammatory changes and oxidative stress. These comorbidities lead to a novel hypothesis that bipolar disorder is a part of a multi-system inflammatory process [8].

Additionally, schizophrenia has a point prevalence of 3.59/1000 in general population [9]. The mean Canadian rates is 25.9 (S.D. *=* 10.5) per 100,000 [10]. Schizophrenia is also comorbid with medical conditions such as diabetes (adjusted OR: 2.11 (1.36 to 3.28) [11], overweight or obesity (44%) [12] and cardiovascular events [13]. Schizophrenia as well as bipolar disorder are part of a multisystem inflammatory processes [14] and anti-inflammatory therapy for treating schizophrenia is suggested [15].

### Mitochondria and bipolar mood disorder

Mitochondria are necessary for the generation of energy and synaptic signaling. Mitochondrial dysfunction appears to be involved in the pathophysiology of bipolar disorder [16]. The prevalence of bipolar disorder in patients with mitochondrial cytopathies is higher than among healthy controls [17]. Reduction in complex one activity of the mitochondrial electron transport chain is documented [18], and there are intriguing preliminary electron microscopy reports of mitochondrial ultrastructural changes in the disorder, particularly abnormal peripheral clustering of mitochondrial in the cytosol [19]. Mitochondrial DNA deletions are increased [20]. Mitochondrial dysfunction has thus been hypothesizes as a molecular basis of bipolar disorder [21]. It is suggested that novel therapeutic agents for treating bipolar mood disorder should target mitochondrial function [16].

### Bipolar disorder and oxidative stress

The generation of oxidatively generated free radicals is core to life, and is normally tightly controlled. However, there is now increasing data on the presence and impact of oxidative stress among diverse psychiatric disorders including bipolar disorder [22], schizophrenia, and autism [23,24]. Reactive oxygen species (ROS) production and metabolism appears to play an important role in the pathophysiology of bipolar disorder [22,25,26]. Not only are both total glutathione and reduced glutathione levels, which are core parts of the intrinsic anti-oxidative system decreased in bipolar disorder, but antioxidant enzyme activities are also impaired [22]. Levels of key redox enzymes including SOD and catalase have been reported to be lower in patients with bipolar disorder than in matched controls [27]. Nitrous oxide (NO) is implicated in the generation of psychotic symptoms such as delusions in bipolar disorder [28]. A meta-analysis reported that the level of NO appears to be significantly increased in bipolar disorder [29]. Moreover, the level of thiobarbituric acidic reactive substances (TBARS), a marker of lipid peroxidation through reaction between free radicals and lipid structures, is increased during manic episodes and remission [30,31]. This finding suggests that oxidative damage to lipid structures is probably continued during the course of bipolar disorder.

### Mechanism of action of established therapies

Lithium is arguably the most effective medication for the prevention of long-term relapse in bipolar mood disorder [32,33]. Lithium’s effects extend beyond its mode of action of monoamine receptors [34]. Chronic treatment with lithium leads to mitochondrial proteins phosphorylation in the rat prefrontal cortex [34]. Lithium also has effects through its impact on inflammation and by the prevention of oxidative stress and cytokine changes [35]. These anti-inflammatory and antioxidative effects are potentially neuroprotective [36,37]. Lithium increases mitochondrial respiratory chain enzyme activities, particularly complex 1, that may be linked to its therapeutic efficacy [38]. Lithium increases mitochondrial energy production [38]. Decreased Na(+)-K(+)-ATPase activity and increased lipid peroxidation in that are seen in bipolar disorder may improve following lithium administration [39]. Long term lithium administration enhances anti-oxidative defenses in bipolar disorder [40] as well as in healthy individuals [41]. N-acetyl-cysteine (NAC), a glutathione based redox modulator, anti-inflammatory agent and mitochondrial modulator [42] decreases symptoms of depression and mania [43] in bipolar disorder [44]. However, established agents have tolerability issues and efficacy limitations, therefore more novel therapeutic approached are needed.

### Mitochondria and schizophrenia

Mitochondrial dysfunction in schizophrenia is frequently reported [45]. Moreover, mitochondrial disorders can present with psychosis [46]. mtDNA plays a role in the neurobiology of schizophrenia [47]. Mitochondrial gene expression is changed in schizophrenia [48]. The numbers of mitochondria in schizophrenia is reduced compared to normal controls [49]. This change in mitochondria may be associated with differential responsiveness to treatment [50]. However, it is not clear whether this number is adaptive or an etiological link to this disorder [51]. Moreover, mitochondrial energy production is impaired in autism [52].

### Schizophrenia and oxidative stress

The role oxidative stress in the neurobiology of schizophrenia is a promising target in order to provide new therapeutic interventions [53]. This is grounded on data that the antioxidant defense system is impaired in schizophrenia [54]. In comparison to normal controls, the activities of superoxide dismutase (SOD), glutathione peroxidase (GSH-Px) are decreased while the levels of MDA are increased chronic schizophrenia [55]. Reduced cellular respiration and complex I abnormalities in schizophrenia are a possible endophenotypic biomarker for schizophrenia [56]. Furthermore, the severity of neurological soft signs in patients with schizophrenia is associated with the level of decreased superoxide dismutase activity [57]. Treatment refractory schizophrenia is associated with higher lipid peroxidation [58].

### Mechanism of action of antipsychotics

The increase of plasma lipid peroxidation in schizophrenia is not due to second-generation antipsychotics [59]. But both typical and atypical antipsychotic medication partially normalize abnormal free radical metabolism in schizophrenia [60]. Long term treatments with both typical and atypical antipsychotics have effects on antioxidant enzymes and lipid peroxidation in schizophrenia [55]. Supplementation of vitamin C with atypical decreases oxidative stress in schizophrenia [61]. N actylcysteine, a redox modulator and glutamate modulator, reduces core symptoms and akathisia in schizophrenia, providing a preliminary proof of concept for the role of oxidative pathways [62].

### Pharmacokinetics of Hydrogen

Molecular hydrogen is an odourless and tasteless gas that has the ability to rapidly diffuse through lipid membranes and enter the cell, where it easily penetrates organelles such as mitochondria as well as the nucleus. It is inert at room temperature and in the absence of catalysts. It additionally easily crosses the blood brain barrier, which facilitates access to the target organs and subcellular components. Its adverse event profile is reportedly benign [2,63]. Physiologically, hydrogen is produced by intestinal microbiota from fermentation of complex carbohydrates. Arterial blood contains higher levels of hydrogen that tissue, suggesting some uptake and utilization of hydrogen in tissues. Hydrogen reacts with free hyrodoxyl radicals but does not appear to react to other reactive oxygen species. This is a theoretical advantage, as low levels of these radicals have physiologically relevant signaling effects. It protects against secondary oxidative damage to the brain in a variety of models by reacting with hydroxyl radicals [64]. Hydrogen is potentially available as a medical gas, hydrogen enriched water, taking a hydrogen bath, injecting hydrogen saline, hydrogen saline eye drops, and augmenting bacterial production of intestinal hydrogen. It penetrates glass but not aluminum containers.

### Hydrogen and oxidative stress and inflammation

Anti-inflammatory effects of hydrogen molecule have been reported in both animal and human models. Hydrogen-rich saline decreases the levels of cytokines IL-4, IL-5, IL-13 and TNF-α in bronchoalveolar lavage fluid [65]. Its effects on tumor necrosis factor alpha, interleukin (IL)-1β and IL-6 levels is hypothesized as the reason for its protective role against UVB radiation [66]. Hydrogen molecule also stops TNFα-induced activation of the NFκB pathway [67]. Hydrogen released by intestinal microbiota, such a hydrogen producing strain of E. coli can suppress inflammation [68]. Taken together, these findings are germane to bipolar disorder as current evidences implicates many interleukins in the neurobiology of bipolar disorder [69].

### Preclinical findings

Inhalation of hydrogen decreased acute lung inflammation in an animal model of acute lung injury [70,71]. Hydrogen enriched water decreased the production of reactive oxygen species in the rat kidney [72]. It protects mitochondria and nuclear DNA from hydroxyl radicals, preventing the decline in mitochondrial membrane potential after antimycin treatment. It has potential in preventing ionizing radiation induced damage, as hydroxyl radicals are the major vehicle for secondary damage. Hydrogen reduces infarction size in a model of middle cerebral artery occlusion and reduced oxidative stress markers [73]. In asphyxiated newborn piglets, it is similarly neuroprotective and preserves cerebrovascular reactivity [74].

### Clinical findings

Hydrogen is hypothesized as a potential therapy for different oxidative stress related diseases such as Parkinson's disease; ischemia/reperfusion of spinal cord, heart, lung, liver, kidney, and intestine; transplantation of lung, heart, kidney, and intestine [75], and autism [76,77]. Adequate hydration with hydrogen-rich water decreases blood lactate levels and improved function of muscle in athletes [78].

Among participants with type 2 diabetes, hydrogen reduced levels of oxidative stress markers [68], and similar findings are reported in the metabolic syndrome [79]. In Parkinson’s disease, increased oxidative stress indexed by elevated lipid peroxidation and decreased reduced glutathione levels in the substantia nigra are part of the known pathogenesis of PD. Hydrogen water also prevents a rat model of Parkinson’s disease [80] and increases survival after cerebral ischemia/reperfusion [81,82]. It down-regulates 4-hydroxy-2-nonenal, a marker of oxidative stress in dopaminergic neurons within the substantia nigra of animal models of Parkinson’s disease. A preclinical study failed to find any association between response to hydrogen and dose [37]. In a pilot placebo controlled, randomized, double-blind, parallel-group design (N = 18), the efficacy of 1000ml molecular hydrogen daily was investigated in patients taking levodopa. In the hydrogen group, there was a reduction in Total Unified Parkinson’s Disease Rating Scale scores, whereas the placebo group deteriorated [83]. Signal modulating activities of hydrogen may play a role in exerting a protective effect against Parkinson’s [79].

It is not fully understood how hydrogen plays its putative antioxidative or anti-inflammatory role. However, it may regulate particular metalloproteins [84]. Moreover, hydrogen reduces the production of peroxynitrite derived from nitric oxide [85]. In patients with rheumatoid arthritis, drinking of water enriched with hydrogen was shown to decreases oxidative stress through scavenging hydroxyl radical and also decreases clinical symptoms after 4 weeks [86]. Moreover, hydrogen-enriched water improves quality of life of patients with liver cancer after radiation exposure [87].

Intravenous administration of 500 ml of H2 enriched fluid has also been examined and it decreased the symptoms of 4 patients with acute erythematous skin diseases with fever and/or pain [88]. A randomized controlled trial indicated that hydrogen-enriched water decreased mitochondrial dysfunction and inflammation in patients with mitochondrial myopathies [89].

Caveats are however necessary, inasmuch as orally administered hydrogen-enriched water may not have enough molecular hydrogen to adequately scavenge hydroxyl radicals. Secondly, the dwell time of hydrogen in the body may be too short to scavenge substantial quantities of continuously generated hydroxyl radicals [90]. In addition, the optimal frequency, dosage, and its method of administration for different diseases are not studied [75].

## Conclusions

Hydrogen gas has a number of biological properties that make it an appealing candidate agent for a diversity of disorders sharing inflammatory, oxidative and apoptotic mechanisms. However, a number of caveats are necessary. Administration is complex, aggravated by its very short biological half-life and low saturation point of 0,8 mM. Furthermore, the low dose used in many studies (0.04-0.08 mM) is shadowed by the fact that intestinal microbiota produce up to 1L a day of hydrogen [91]. There is currently very a limited understanding of the pathways and processes impacted by hydrogen. Greater preclinical data is required to elucidate the biological mechanisms of hydrogen. It however is true that many agents are in widespread use despite poor understanding of their mechanisms of action, particularly in the neurosciences. This therefore does not preclude use, which is therefore far more contingent on safety issues. Given that hydrogen does seem to have a benign safety profile, albeit based on very limited data, moving to clinical studies appears warranted. We additionally need a more complete understanding of hydrogen’s dosage, mode of administration, pharmacokinetics, biology and toxicity of hydrogen to facilitate clinical application. Nevertheless, there is much that is appealing about this avenue, and it merits greater investment given the paucity of therapeutic options and their limitations.

## Competing interests

AG has no related conflict of interest to be declared. MB has received Grant/Research Support from the NIH, Cooperative Research Centre, Simons Autism Foundation, Cancer Council of Victoria, Stanley Medical Research Foundation, MBF, NHMRC, Beyond Blue, Rotary Health, Geelong Medical Research Foundation, Bristol Myers Squibb, Eli Lilly, Glaxo SmithKline, Organon, Novartis, Mayne Pharma and Servier, has been a speaker for Astra Zeneca, Bristol Myers Squibb, Eli Lilly, Glaxo SmithKline, Janssen Cilag, Lundbeck, Merck, Pfizer, Sanofi Synthelabo, Servier, Solvay and Wyeth, and served as a consultant to Astra Zeneca, Bristol Myers Squibb, Eli Lilly, Glaxo SmithKline, Janssen Cilag, Lundbeck Merck and Servier, and is a co-inventor of two provisional patents regarding the use of NAC and related compounds for psychiatric indications, which, while assigned to the Mental Health Research Institute, could lead to personal remuneration upon a commercialization event.

## Authors’ contributions

AG was responsible for the initial conception and drafting of the manuscript. AG and MB revised the preliminary draft of the manuscript. Both authors read and approved the final manuscript.
